# Effects of Betaine on LPS-Stimulated Activation of Microglial M1/M2 Phenotypes by Suppressing TLR4/NF-κB Pathways in N9 Cells

**DOI:** 10.3390/molecules24020367

**Published:** 2019-01-21

**Authors:** Hui Shi, Xiao-Long Wang, Hong-Feng Quan, Lin Yan, Xiu-Ying Pei, Rui Wang, Xiao-Dong Peng

**Affiliations:** 1 Department of Pharmacology, Ningxia Medical University, Yinchuan 750004, China; 15771377892@163.com (H.S.); nomoredrama@126.com (X.-L.W.); quanhongfeng@163.com (H.-F.Q.); 2 Functional Experiment Center, School of Basic Medicine, Ningxia Medical University, Yinchuan 750004, China; Yanlin1@Tom.com; 3 Laboratory in Fertility Preservation and Maintenance of Ministry of Education, Ningxia Medical University, Yinchuan 750004, China; peixy@nxmu.edu.cn

**Keywords:** betaine, microglia, M1/M2 polarisation, N9 microglial cells, neuroinflammation

## Abstract

Microglia mediate multiple facets of neuroinflammation. They can be phenotypically divided into a classical phenotype (pro-inflammatory, M1) or an alternative phenotype (anti-inflammatory, M2) with different physiological characteristics and biological functions in the inflammatory process. Betaine has been shown to exert anti-inflammatory effects. In this study, we aimed to verify the anti-inflammatory effects of betaine and elucidate its possible molecular mechanisms of action in vitro. Lipopolysaccharide (LPS)-activated microglial cells were used as an inflammatory model to study the anti-inflammatory efficacy of betaine and explore its mechanism of regulating microglial polarisation by investigating the morphological changes and associated inflammatory changes. Cytokine and inflammatory mediator expression was also measured by ELISA, flow cytometry, immunofluorescence, and western blot analysis. Toll-like receptor (TLR)-myeloid differentiation factor 88 (Myd88)-nuclear factor-kappa B (NF-κB) p65, p-NF-κB p65, IκB, p-IκB, IκB kinase (IKK), and p-IKK expression was determined by western blot analysis. Betaine significantly mitigated the production of pro-inflammatory cytokines and increased the release of anti-inflammatory cytokines. It promoted the conversion of the microglia from M1 to M2 phenotype by decreasing the expression of inducible nitric oxide synthase and CD16/32 and by increasing that of CD206 and arginase-1. Betaine treatment inhibited the TLR4/NF-κB pathways by attenuating the expression of TLR4-Myd88 and blocking the phosphorylation of IκB and IKK. In conclusion, betaine could significantly alleviate LPS-induced inflammation by regulating the polarisation of microglial phenotype; thus, it might be an effective therapeutic agent for neurological disorders.

## 1. Introduction

Neuroinflammation is an ineluctable and significant pathological process involved in all types of damages and disorders of the central nervous system (CNS) [1]. Microglia, which belong to the non-neuronal glial system, are resident mononuclear phagocytes in the CNS [2]. During CNS damage because of infections, brain trauma, or ischaemic injury, microglia change from the resting state to an amoebic activated state [3]. Activated microglia can be classified into two main types: the classical (M1 type) and alternative (M2 type) activation states. Pro-inflammatory cytokines interferon, tumour necrosis factor (TNF), and lipopolysaccharide (LPS) can activate microglia typically [4]. Activated M1 microglia can produce related factors, such as pro-inflammatory cytokines and inflammatory mediator, which elicit toxic effects on neurons and promote tissue inflammation and damage. After M1 polarisation, these cells often enter the repair phase. By contrast, M2-type microglia secrete anti-inflammatory mediators and neurotrophic factors, such as transforming growth factor-β [5,6]. M1 and M2 phenotypes, the different ‘activated’ states of microglia, represent only two extremes of a series of activation state spectra of the microglia rather than a single cell phenotype, which determines their respective physiological characteristics and different biological functions [7]. It stands to reason that during neuroinflammation-related disease, such as neurodegenerative disease and major depression where neuroinflammation is a prominent feature and potential contributor to disease, the pharmacological compounds alternatively activated microglia would be the new alternative beneficial in resolving pathology.

The toll-like receptor (TLR) 4/myeloid differentiation factor 88 (Myd88)/nuclear factor-κB (NF-κB) pathways are critical to molecular modulation. Current evidence suggests that TLR4 is the only known member of the TLR family that engages all the four toll-interleukin receptor (TIR) domain-containing adaptor proteins to signal the inflammatory response [8]. Stimulation of TLR4 can further activate the NF-κB pathway [9]. NF-κB is expressed in almost all cells, and plays an important role in the activation of M1-phenotype microglia [10]. NF-κB is the main activator of microglia-mediated inflammatory cytokine release, which is the basis of future research.

Inhibition of the M1 and promotion of M2 phenotype are potentially more feasible approaches for controlling neuroinflammation than merely inhibition of M1 activation only. However, pharmacological compounds have been shown to regulate the transformation of microglia to the M2 phenotype are rare [11,12,13]. Therefore, drug-induced polarisation of the microglia from the M1 phenotype to the M2 phenotype can provide a new strategy for the treatment of neuroinflammation disorders.

Betaine, also known as trimethylglycine, is a water-soluble compound that penetrates the blood-brain barrier and exhibits high distribution to the hippocampus in vitro [14,15]. Betaine has shown various pharmacological effects, such as antiepileptic, neuroprotective, and memory-improving effects [14,16,17,18]. In particular, it acts as a methyl donor to interfere with depression caused by hyperhomocysteinemia [19]. Additionally, wolfberry (*Lycium barbarum*, *Solanaceae*), a traditional food and folk medicinal plant in China and East Asia, is a rich source of betaine. Betaine was shown to exert anti-inflammatory effects [20,21]. However, whether betaine could inhibit the polarisation of LPS-induced microglial phenotype remains unclear. Moreover, the possible molecular mechanisms of betaine actions need to be elucidated. In the current study, we hypothesised that betaine could regulate LPS-induced activation of the microglia via the TLR4/NF-κB signalling pathway. LPS-activated N9 microglial cells were used as an inflammatory model to study the anti-inflammatory efficacy of betaine by investigating the morphological and inflammatory changes. Inflammatory mediator and cytokines, such as nitric oxide (NO), interleukin (IL)-6, IL-1β, IL-10 and tumor necrosis factor-α (TNF-α) were also investigated. In addition, we measured the expression of proteins involved in the polarisation of microglia to determine the relationship between the anti-inflammatory effects and phenotypic changes. Additionally, we examined whether betaine exerted its anti-inflammatory effects by suppressing the NF-κB pathway.

## 2. Results

### 2.1. The Effects of Betaine on the Viability and Morphological Changes of N9 Microglial Cells Treated with LPS

**Figure 1:**
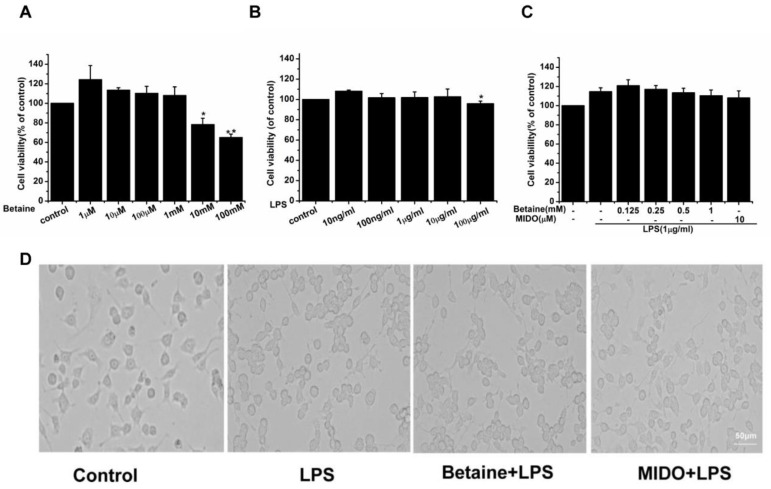
Effects of betaine on the viability and morphological changes of N9 microglial cells with or without LPS stimulation. (**A**) Effects of betaine alone on cell viability. (**B**) Effects of lipopolysaccharide (LPS) alone on cell viability. (**C**) Effects of betaine with or without LPS stimulation on cell viability. Cells were treated with betaine or MIDO (10 μM) for 1 h and then stimulated with LPS (1 μg/mL) for 24 h. (**D**) Effects of betaine with or without LPS stimulation on morphological changes. MIDO was used as a positive control. Data are presented as the means ± SEM of three independent experiments. Untreated cells served as a control group. * *p* < 0.05 and ** *p* < 0.01, compared to the control group.

The survival rate of N9 microglial cells at different concentrations of betaine and LPS was tested using the cell counting kit-8(CCK-8) assay. To estimate the range of effective concentrations, we first measured the effects of betaine on the viability of N9 microglial cells at concentrations ranging from 1 μM to 100 mM. Results (Figure 1A) showed that betaine at concentrations ˂ 10 mM alone did not cause any detectable cytotoxicity in N9 microglial cells. Then, the effects of LPS alone at concentrations ranging from 10 ng/mL to 100 μg/mL on the viability of N9 microglial cells were investigated. Results showed (Figure 1B) that LPS alone stimulated N9 microglial cells and compromised cytotoxicity at a concentration of 100 μg/mL, whereas it did not induce microglial cell viability up to 10 μg/mL. N9 microglial cells was pretreated with different concentrations of betaine or minocycline (MIDO, 10μM) for 1 h and then incubated for 24 h with or without LPS. MIDO was used as a positive control. Betaine at different concentrations (0.125, 0.25, 0.5, and 1 mM) did not induce N9 microglial cell death (Figure 1C). Therefore, the non-toxic concentrations of betaine (1 mM) and LPS (1 μg/mL) were selected for the subsequent experiments. Morphological changes in N9 microglial cells were evaluated after treatment with betaine (1 mM) or MIDO (10 μM) with or without LPS. As shown in Figure 1D, resting microglia exhibited small cell bodies and elongated branching. After treatment with LPS, the microglia acquired a proinflammatory M1 morphology, characterised by thick and short cell bodies. However, LPS-induced morphological changes in N9 microglial cells were attenuated after treatment with betaine or MIDO.

### 2.2. Effects of Betaine on the Production of NO and Inflammatory Cytokines in LPS-Induced N9 Microglial Cells

**Figure 2:**
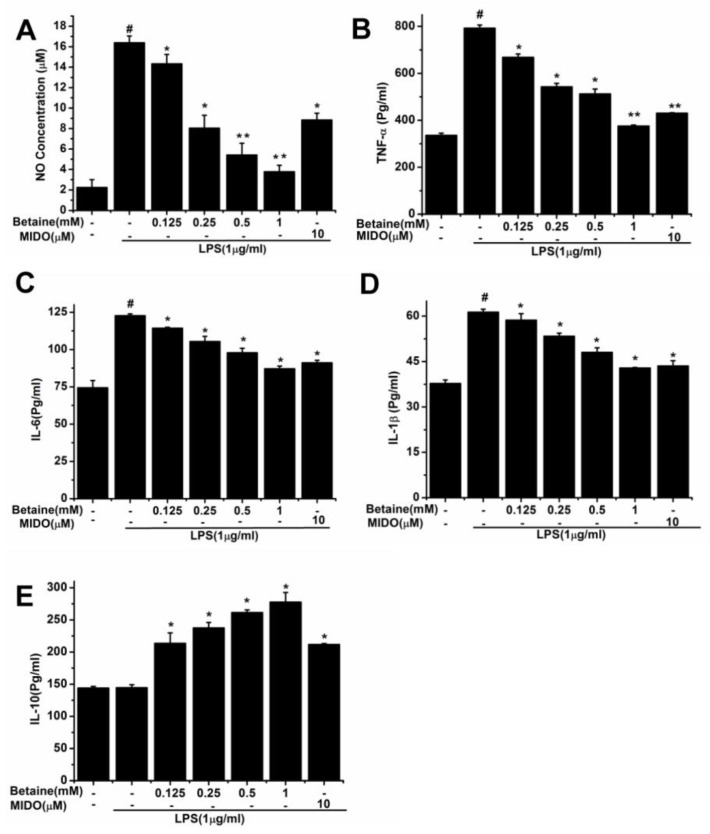
Effects of betaine on LPS-induced inflammatory cytokine and NO release in N9 microglial cells. Cells were treated with betaine or MIDO (10 μM) for 1 h and then incubated with or without LPS (1 μg/mL) for 24 h. (**A**) NO concentration in the supernatants was measured by NO one-step detection kit. (**B**–**E**) Levels of TNF-α, IL-6, IL-1β, and IL-10 in the supernatants were determined by ELISA. MIDO was used as a positive control. Data are presented as the means ± SEM of three independent experiments. The control group included untreated cells. Untreated cells served as a control group. # *p* < 0.05, compared to the control group; * *p* < 0.05 and ** *p* < 0.01, compared to the LPS-treated group.

N9 microglial cells was pretreated with different concentrations of betaine or MIDO (10 μM) for 1 h and then incubated for 24 h with or without LPS. To assess the effects of betaine on LPS-induced inflammatory mediators, we first evaluated the production of NO. Results showed (Figure 2A) that NO level considerably increased after LPS treatment, compared to that in the control group. Importantly, betaine (0.125–1 mM) reduced NO levels in a dose-dependent manner. LPS-induced production of TNF-α, IL-6, IL-1β, and IL-10 was measured by ELISA. Results showed (Figure 2B–D) that M1 proinflammatory polarisation of N9 microglial cells greatly increased after LPS stimulation, as evidence by the production of M1 proinflammatory cytokines (TNF-α, IL-6, and IL-1β). The M2 anti-inflammatory cytokine (IL-10) was not markedly changed after LPS stimulation (Figure 2E). Interestingly, LPS-induced M1 proinflammatory cytokine (TNF-α, IL-6, and IL-1β) production was inhibited in a dose-dependent manner after betaine (0.125–1 mM) treatment (Figure 2B–D). In contrast, betaine (0.125–1 mM) increased the production of M2 anti-inflammatory cytokine (IL-10) in a dose-dependent manner (Figure 2E). These results indicated that betaine exhibited anti-inflammatory effects in LPS-stimulated N9 cells. Moreover, betaine at 1 mM was further used in subsequent experiments. MIDO was used as a positive control.

### 2.3. Effects of Betaine on LPS-Induced Expression of CD16/32 and CD206 Proteins in N9 Microglial Cells

**Figure 3:**
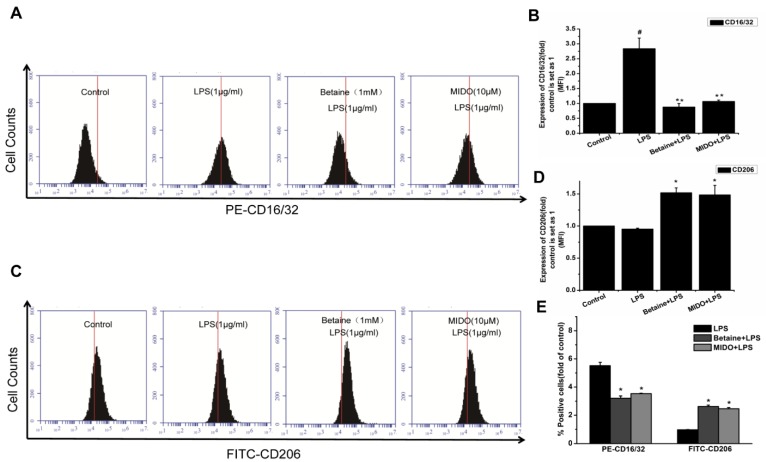
Effects of betaine on LPS-induced protein expression of CD16/32 and CD206 in N9 microglial cells. N9 microglial cells was treated with betaine (1 mM) or MIDO (10 μM) for 1 h and then incubated with or without LPS (1 μg/mL) for 24 h. (**A**,**C**) CD16/32 (M1) and CD206 (M2) protein expression levels were determined by flow cytometry. (**B**,**D**) The expression levels of CD16/32 and CD206 with or without LPS treatment were compared. Control is set as 1. (**E**) Quantitative positive cells of an overlay of “control” with each of the treatments. MIDO was used as a positive control. Data are presented as the means ± SEM of three independent experiments. The control group included untreated cells. Untreated cells served as a control group. # *p* < 0.05, compared to the control group; * *p* < 0.05 and ** *p* < 0.01, compared to the LPS-treated group.

CD16/32 and CD206 are specific membrane proteins and M1 and M2 polarisation markers, respectively. We measured CD16/32 and CD206 expression by flow cytometry to determine the effect of betaine on N9 microglial cell polarisation. Figure 3A and B show that the expression of the M1 polarisation marker, CD16/32 was significantly lower after betaine (1 mM) pretreatment than that in the LPS group. The expression of CD206 (M2 marker) markedly increased in betaine-pretreated N9 microglial cells, compared to that in the LPS group (Figure 3C,D). MIDO was used as a positive control.

### 2.4. Betaine Promoted Microglial Polarisation to the M2 Phenotype in LPS-Induced N9 Microglial Cells

**Figure 4:**
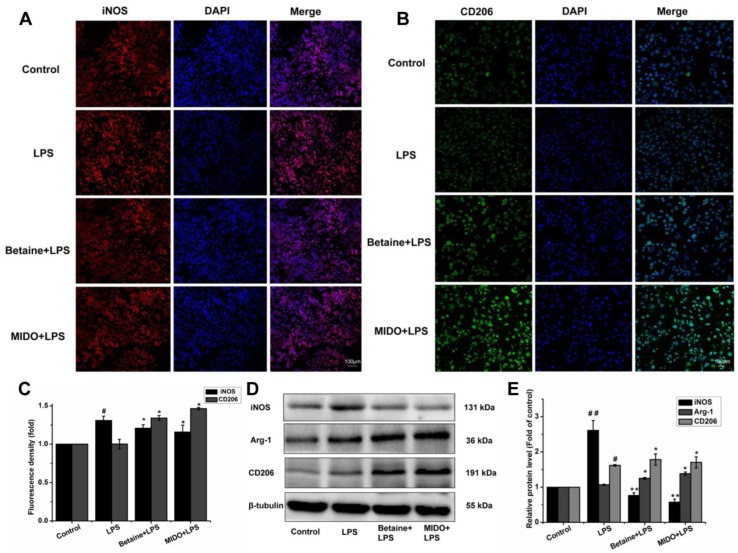
Betaine promoted microglial polarisation to the M2 phenotype in LPS-induced N9 microglial cells. Cells were treated with betaine (1 mM) or MIDO (10 μM) for 1 h and then incubated with or without LPS (1 μg/mL) for 24 h. (**A**,**B**) Cells were counterstained with anti-iNOS (M1 marker, red) and anti-CD206 (M2 marker, green) antibodies. Nuclei were stained with DAPI (blue), and fluorescence was observed by confocal microscopy. (**C**) Representative images of the fluorescence intensity of iNOS and CD206 with or without LPS treatment. (**D**,**E**) Expression of iNOS, Arg-1, and CD206 proteins was analysed by western blot analysis. MIDO was used as a positive control. Data are presented as the means ± SEM of three independent experiments. The control group included untreated cells. Untreated cells served as a control group. # *p* < 0.05 and ## *p* < 0.01 compared to the control group; * *p* < 0.05 and ** *p* < 0.01, compared to the LPS-treated group.

To further determine whether betaine switches the polarisation of N9 microglial cells to the anti-inflammatory M2 phenotype under LPS stimulation, nitric oxide synthase (iNOS), arginase-1 (Arg-1), and CD206 expression was measured. iNOS represents M1 microglial polarisation, whereas Arg-1 and CD206 represent M2 microglial polarisation. Immunofluorescence staining (Figure 4A,B) and western blot analysis (Figure 4D,E) showed that LPS-stimulated N9 microglial cells exhibited higher expression of iNOS than that in the control (Figure 4A); however, the expression of CD206 in N9 cells was not markedly changed by LPS (Figure 4B). Furthermore, the expression of Arg-1 slightly increased (Figure 4D). Interestingly, betaine significantly decreased the expression of iNOS and increased that of CD206 and Arg-1 in LPS-stimulated N9 cells. MIDO was used as a positive control. In summary, these results indicated that betaine shifted LPS-induced inflammatory M1 microglia toward anti-inflammatory M2 microglia.

### 2.5. Effects of Betaine on LPS-Induced TLR4/NF-κB Pathways in N9 Microglial Cells

**Figure 5:**
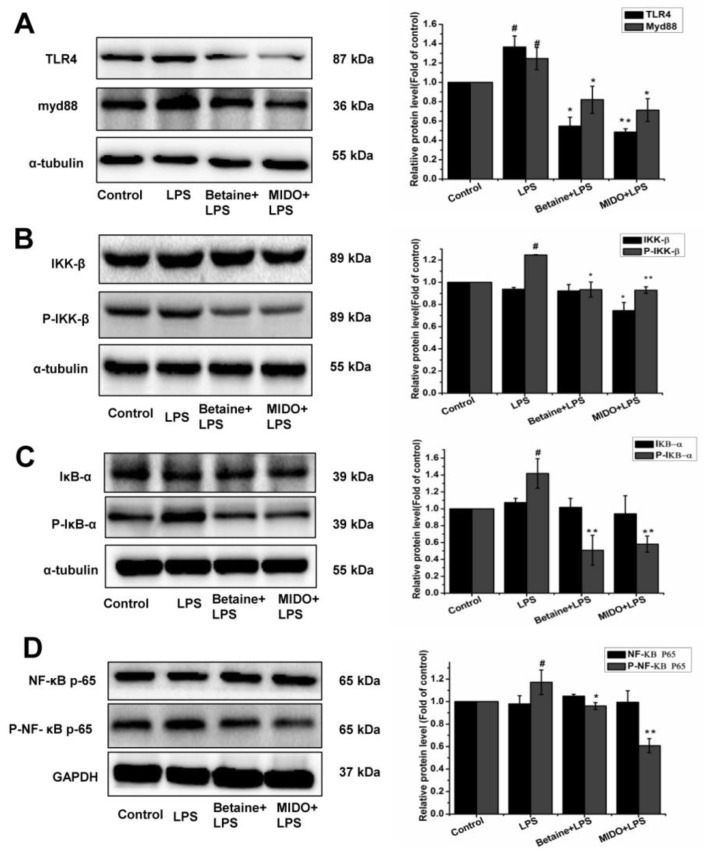
Effects of betaine on LPS-induced TLR4/NF-κΒ signal transduction in N9 microglia cells. Cells were treated with betaine (1 mM) or MIDO (10 μM) for 1 h and then incubated with or without LPS (1 μg/mL) for 24 h. (**A**) Determination of TLR4/Myd88 protein levels by western blot analysis. (**B**–**D**) Determination of IKK-β, P-IKK-β, IκΒ-α, P-IκΒ-α, NF-κΒ, and p-NF-κΒ protein levels by western blot analysis. MIDO was used as a positive control. Data are presented as the means ± SEM of three independent experiments. The control group included untreated cells. Untreated cells served as a control group. # *p* < 0.05, compared to the control group; * *p* < 0.05 and ** *p* < 0.01, compared to the LPS-treated group.

TLR4 plays a key role in the development of innate defence mechanisms and adaptive immunity against pathogens, and Myd88 acts as an intermediate linker for inflammatory motility [1]. Compared to the control group (Figure 5A), LPS stimulation significantly upregulated TLR4 and Myd88 protein expression, indicating that LPS induced TLR4 and Myd88 activation. However, betaine treatment effectively inhibited TLR4 and Myd88 protein expression in N9 microglial cells. Given that Myd88 signalling mediates the NF-κΒ pathway, we evaluated the activation of the NF-κΒ pathway by western blot analysis. As shown in Figure 5B–D, LPS stimulation resulted in the phosphorylation of IκB kinase (IKK-β), IκB-α, and NF-κΒ p65 but did not affecting IKK-β, IκΒ-α and NF-κΒp-65 their activity expression. However, betaine treatment decreased the expression of p-IKK-β, p-IκΒ-α, and p-NF-κB p65. These results indicated that betaine inhibited the LPS-induced activation of the TLR4/NF-κΒ pathways in N9 microglial cells in vitro. MIDO was used as a positive control.

## 3. Discussion

Neuroinflammation is accompanied by the activation of microglia and the release of proinflammatory cytokines [2,22]. Microglia account for 10–15% of the total cell population in the brain parenchyma and exhibit a neurospecific phenotype during activation [23]. Besides the quiescent state, two functionally different activation states exist, namely, M1 and M2 [24]. Distinct polarisation options yield microglia with diversified functions, some of which are beneficial, whereas others are harmful for neurons in the CNS. Most of the compounds that reduce neuroinflammation only inhibit M1-phenotype microglia. Few compounds have been shown to promote the polarisation of microglia to the M2 phenotype [25,26]. It has been suggested that microglia activation states in neuroinflammation may need to be specifically treated by simultaneously attenuating M1 phenotype and promoting M2 phenotype responses [27]. But characterization of the repair-promoting M2-phenotypes is relatively complex [28]. For instance, in the early stage of cerebral ischemia in ICH rats [29], local administration of IL-4 was suggested to be associated with a diminished M2 alternative phenotype. But, on the other hand, one study described an increase in amyloid pathology possibly due to IL-4 inhibiting microglia from properly scavenging Aβ in Alzheimer’s disease mouse model [30]. An either M1 or M2 phenotype activation state of microglia is beneficial in resolving pathology in neuroinflammation-related disease still remains unclear. Therefore, microglia transition from a M1 responses to a more regulatory M2 responses depending upon different environmental [7,28]. Alternatively, drugs that can induce shifting of the microglia from the M1 to M2 phenotype might useful in alleviating subsequent brain parenchymal damage. Glatiramer acetate [31] and β-interferon [25] not only suppressed M1 reactions but also promoted the balance between M1 and M2 microglial cells. Likewise, pharmacological compounds have been shown to regulate the transformation of microglia to the M2 phenotype [1,11,12]. Betaine (*N*-trimethylglycine) not only serves as an metabolic intermediate and permeate but also may affect pathways involved in suppressing neurotransmitter production and reuptake [32]. The role of betaine in the nervous system has increasingly gained attention. It has been shown to improve cognition and confer neuroprotection [14,17]. Importantly, betaine exhibited anti-inflammatory effects, was involved in the inflammatory response to local hyperosmosis, and effectively inhibited the production of NO in LPS-induced microglia [20,21]. Therefore, results of our study verified the anti-inflammatory effects of betaine and showed its relationship with the phenotypic changes.

Several studies investigated the use of MIDO as a means to repress M1 microglial activation and provide neuroprotection [33,34,35]. Therefore, we used MIDO as a positive control and verified its ability to inhibit microglial polarisation into the anti-inflammatory M2 type. The findings of our study indicated that betaine exerted comparable anti-inflammatory effects to those of MIDO, which are in line with the results of previous studies [35,36,37].

LPS is a typical TLT4 agonist that not only polarises the microglia into the M1 pro-inflammatory phenotype but also induces inflammatory responses and reduces the expression of M2 anti-inflammatory markers, thereby inducing inflammation [5,38]. Therefore, LPS-stimulated microglia were used to study the anti-inflammatory efficacy of betaine by exploring the mechanism of regulation of microglial polarisation. We showed that betaine did not compromise microglial cell viability at a wide range of biocompatible concentrations (up to 1 mM). Resting microglia exhibited small cell bodies and elongated branches. When a dangerous signal was encountered, such as LPS treatment, phagocytic cells acquired a pro-inflammatory M1 phenotype that is characterised by hypertrophic bodies with short, few and thick processes [28]. In our study, most of the LPS-induced microglial cells exhibited morphological changes toward the activated phenotype, whereas betaine attenuated LPS-induced morphological changes in N9 microglial cells.

Several recent studies characterised the microglia and distinguished between the M1 and M2 phenotypes based on three aspects; inflammation-related cytokines [39], specific cell membrane proteins, and arginine metabolism-associated molecules [40].To further determine whether betaine could induce the polarisation of N9 microglial cells to the anti-inflammatory M2 phenotype under LPS stimulation, NO, TNF-α, IL-1β, IL-6, and IL-10 release were measured to assess inflammation-related factors. Additionally, specific markers were investigated to assess the polarisation of N9 microglia cell: Arg-1 and iNOS as arginine metabolism-related molecules and CD16/32 and CD206 as specific cell membrane proteins.

M1-type microglia exhibit strong phagocytic ability and can produce several proinflammatory factors, including IL-1β, IL-6, TNF-α, and inducible NO synthase (iNOS) [28], thereby promoting inflammatory response and aggravating neuronal damage. M2-type microglia secrete anti-inflammatory mediators, tissue inhibitor of metalloproteinase-1, and Arg-1 after stimulation with IL-4, IL-10, or IL-13 [41]. The anti-inflammatory M2 alternative phenotype presents neuro- protective effects, such as immune regulation, angiogenesis, wound healing, and anti-inflammatory effects by promoting anti-inflammatory factor release and inactivating proinflammatory cell phenotypes [13]. For instance, M2-phenotype microglia induce IL-10 production to re-establish homeostasis [42]. Our results showed that betaine exerted anti-inflammatory effects in the LPS-stimulated N9 microglial cells, as evidenced by the inhibition of LPS-induced production of M1 proinflammatory cytokines, such as TNF-α, IL-1β, and IL-6 in a dose-dependent manner. Conversely, betaine promoted the release of IL-10 by the activated microglia in a dose-dependent fashion.

Moreover, studies on the microglia are no longer limited to inhibition of neuroinflammation and neurodegenerative diseases, but they focus on simultaneously attenuating the M1 responses and promoting the M2 responses [43]. Thus, we evaluated whether betaine could shift LPS-activated M1 phenotype to M2 phenotype. It has been shown that excessive NO contributes to neuronal damage, and betaine could decrease NO level to control neurological disorders [44]. In line with the results of previous studies showing that betaine strongly inhibited NO production in LPS-stimulated microglial cells [20]. Our data indicated that betaine decreased NO production in a dose-dependent manner after 24 h. In addition, M1 microglia likely produce cytotoxic NO via iNOS, the main microglial enzyme responsible for NO production [41]. Accordingly, we measured the release of NO produced by N9 microglial cells in the absence or presence of LPS and tested whether betaine affected iNOS expression. Betaine prevented NO production and inhibited iNOS expression, thus repressing the M1 phenotype. Furthermore, the expression of anti-inflammatory mediators of the M2a-repair/regeneration phenotype (namely, Arg-1) has been shown to be consistent with a repair phenotype in response to IL-4 or IL-10 [45], expression of this marker is consistent with a repair reparatory phenotype [46], which suggests that alternatively activated microglias play an anti-inflammatory or neuroprotective role [47]. The expression of the M2 marker, Arg-1 increased after betaine treatment, suggesting that betaine might promote an M2 alternative phenotype. Overall, iNOS and Arg-1 were suggested to play important roles in phenotype determination, where iNOS and Arg-1 expression levels affected the inflammatory response in an opposite fashion.

Moreover, CD16/32 and CD206, specific cell membrane proteins, play an important role in phagocytosis and antigen presentation of the microglia [46,48]. CD16/32 acts as an M1 marker, whereas CD206 is an M2 marker. Our data indicated that betaine inhibited the expression of M1 phenotypic markers and promoted the polarisation of microglia to the M2 phenotype.

Multiple signalling pathways are involved in LPS-induced activation of the microglia. Similar to peripheral macrophages, microglia express a complete set of pattern recognition receptors [9]. Both TLR4 and Myd88 signal transduction pathways can activate the downstream IKK complex, resulting in activation of the NF-κB pathway and expression of various innate immune and inflammatory factors. At rest, IκB-α binds to the p50 and p65 subunits of NF-κB. Once microglia are activated, IκB-α is separated and transferred from the cytoplasm to the nucleus, resulting in transcription and expression of inflammatory factors [49]. NF-κB is expressed in nearly all cells and plays an important role in the activation of the M1 microglial phenotype [50]. Our results further showed that betaine interacted with the IKK complex and specifically targeted its IKK-β subunit, thereby protecting the IκB subunit from LPS-mediated inflammatory stimulus-induced phosphorylation and subsequent proteasomal degradation. Therefore, betaine induced polarisation of the microglia to the M2 phenotype and inhibited LPS-induced inflammatory response by possibly inhibiting the TLR4/NF-κB pathways.

## 4. Materials and Methods

### 4.1. Cell Culture and Treatment

N9 microglial cells was obtained from TongPai Biological Technology Co., Ltd. (Shanghai, China) and cultured in Roswell Park Memorial Institute (RPMI)-1640 medium (Gibco, Thermo Fisher Scientific, Inc., Waltham, MA, USA) containing 10% foetal bovine serum (FBS; Gibco, Thermo Fisher Scientific, Inc.), 100 IU/mL penicillin, and 100 μg/mL streptomycin (Beijing Solarbio Science & Technology Co., Ltd., Beijing, China). The cells were incubated at 37 °C in the presence of 5% CO_2_. Betaine (purity > 99%, SLBH8652V), MIDO (WXBC3872V), and LPS (088M4067V) were all purchased from Sigma-Aldrich (St-Louis, MO, USA). Cells were pretreated with betaine or MIDO for 1 h and then treated with or without LPS (1 μg/mL) for 24 h. MIDO was used as a positive control.

### 4.2. Cell Viability Assay and Morphological Analysis

The viability of microglial N9 cells was determined by CCK-8 (Jiangsu KeyGen Biotech Co., Ltd., Nanjing, China) assay. In brief, N9 microglial cells was cultured in 96-well plates with betaine (1 μM–100 mM) and LPS (10 ng/mL–100 μg/mL) for 24 h. The cells were treated with betaine (0.125, 0.25, 0.5 and 1 mM) for 1 h and then treated with or without LPS (1 μg/mL) for 24 h. CCK-8 solution (10 μL) was added, and the plates were incubated at 37 °C for 3 h. The absorbance was measured at 450 nm using a plate reader (Thermo Scientific). For morphological analysis, cells were imaged with a laser scanning confocal microscope (Olympus, Tokyo, Japan) at 40× magnification.

### 4.3. NO Assay

A cell suspension was prepared by inoculating N9 microglial cells in the logarithmic growth phase into a 6-well culture plate. After 24 h, cells were pretreated with different concentrations of betaine (0.125, 0.25, 0.5, and 1 mM) for 1 h, and then incubated with or without LPS (1 μg/mL) for 24 h. The supernatant was collected, and NO production was determined using a NO assay kit (microwell plate method, Nanjing Jiancheng Bioengineering Institute, Nanjing, China). The absorbance of the reaction mixtures was measured at 450 nm using a microplate reader (Thermo Scientific).

### 4.4. ELISA Assay for Determination of Cytokine Levels

A cell suspension was prepared by inoculating N9 microglial cells in the logarithmic growth phase into a 6-well culture plate. TNF-α, IL-1β, IL-6, and IL-10 levels were measured using ELISA kits (XY Biotechnology, Shanghai Xinyu Biotechnology Co., Ltd., Shanghai, China). After 24 h, cells were pretreated with different concentrations of betaine (0.125, 0.25, 0.5, and 1 mM) for 1 h, and then incubated with or without LPS (1 μg/mL) for 24 h. The supernatant was collected, and cytokine levels were measured by ELISA, according to the manufacturer’s instructions. Optical density (OD) was measured at 450 nm using a microplate reader (Thermo Scientific).

### 4.5. Flow Cytometry Analysis

The cells were digested or isolated from the culture dish using trypsin (Beijing Solarbio Science & Technology Co., Ltd., Beijing, China). Then, they were washed with phosphate-buffered saline (PBS) and counted. The cell concentration of each group was detected by flow cytometry, and adjusted to 1 × 10^6^ cells/mL. Approximately 100 μL of the cell suspension was resuspended in a 1-mL Eppendorf tube, and then anti-mouse CD16/32PE (Biosciences, BD, USA) and anti-mouse CD206 FITC (BioLegend, SanDiego, CA, USA) were added at 4 °C and incubated for 0.5 h in the dark. The cells were washed twice with PBS and resuspended in 500 μL of 1× PBS solution. Results were expressed as the mean fluorescence intensity (BD Biosciences, Franklin Lakes, NJ, USA).

### 4.6. Immunofluorescence Staining

A slide coated with poly L-lysine (0.1 mg/mL) was placed in a 24-well plate. N9 microglial cells were cultured and treated with the indicated treatments and fixed with 4% paraformaldehyde for 30 min. The cells were permeabilised with 0.1% Triton X-100 for 20 min and then blocked with PBS containing 2% bovine serum albumin (BSA; Sigma-Aldrich) for 1 h. The cells were incubated with anti-mouse CD206 (1:200, Abcam, Cambridge, MA, USA) and anti-mouse iNOS (1:100, Abcam Cambridge, MA, USA) overnight at 4 °C. On the following day, cells were washed with PBS and then incubated with FITC-labelled goat anti-rabbit IgG (1:200, Proteintech Group, Chicago, IL, USA) and tetramethylrhodamine (TRITC)-conjugated goat anti-rabbit IgG (1:50, Proteintech Group, Chicago, IL, USA) for 2 h. The cells were then subjected to immunofluorescence staining with DAPI for 5 min at 37 °C then examined under a confocal microscope (Olympus) at 20× magnification.

### 4.7. Western Blot Analysis

Cells were lysed by shaking in radioimmunoprecipitation assay (RIPA) lysis buffer supplemented with protease inhibitor (Nanjing Key Gen Biotech Co., Ltd., Nanjing, China). The protein concentration was measured by the bicinchoninic acid (BCA) assay (Beijing Trans Gen Biotech Co., Ltd. Beijing, China). Cell extract (50 μg) was applied to a sodium dodecyl sulphate (SDS)-polyacrylamide gel electrophoresis, transferred to a polyvinylidene fluoride (PVDF) membrane, and then blocked with PBST containing 5% skimmed milk for 2 h. The membrane was incubated at 4 °C with primary antibodies (TLR4, 1:500; Myd88, 1:1000; iNOS, 1:1000; CD206, 1:2000; Arg-1, 1:1000 [Abcam]; IKK-β, 1:500; p-IKK-β, 1:200; IκBα, 1:1000; p-IκBα, 1:1000; NF-κB, 1:1000; p-NF-κB, 1:1000; GAPDH, 1:2000; α-tubulin, 1:1000; and β-tubulin, 1:1000 [Proteintech]) overnight. On the next day, the membrane was washed four times for 8 min each with PBST and incubated with HRP-anti-rabbit secondary antibody (1:5000; Proteintech) for 1 h. The bands were visualised by enhanced chemiluminescence kit (Advansta, Menlo Park, CA, USA), and the results were analysed using Quantity One (Bio-Rad Laboratories, Inc. Hercules, CA, USA) software. GAPDH, α-tubulin, and β-tubulin were used as internal references.

### 4.8. Statistical Analysis

Data analysis was performed using the statistical software SPSS 17.0 (SPSS Inc., USA) and Origin 8.0 software programs (Origin Lab, Northampton, MA, USA). Data were expressed as the means ± standard error (SE). Data were statistically analysed using one-way analysis, followed by Student’s t-test (comparing two groups) or LSD post-hoc test (comparing more than two groups). *p* < 0.05 was considered statistically significant.

### 4.9. Conclusions

In summary, betaine exerted anti-inflammatory effects in the microglia and shifted microglial polarisation to the M2 phenotype by suppressing the TLR4/NF-κB pathways. Therefore, betaine might be an effective therapeutic candidate to mitigate inflammation and promote functional recovery from neurological disorders.
